# Prevalence of Refractive Errors Among Children at a Tertiary Care Center in Karnataka: A Cross-Sectional Study

**DOI:** 10.7759/cureus.62172

**Published:** 2024-06-11

**Authors:** Karishma Munoli, Siddesh Harpanalli, Ramanna Chalvadi, Aishwarya Polisgowdar, Bylappanavara Girish, Garlapati V Vishnu

**Affiliations:** 1 Department of Ophthalmology, Raichur Institute of Medical Sciences, Raichur, IND

**Keywords:** refractive error, prevalence, myopia, hypermetropia, astigmatism

## Abstract

Background: Refractive errors (REs) are the major cause of blindness and impaired vision with considerable morbidity. Finding the prevalence with early detection of REs with appropriate corrective measures can bring down eye morbidity in children.

Aim: The aim of the study was to find the prevalence of REs among children attending Raichur Institute of Medical Sciences Teaching Hospital in Karnataka State of South India.

Methodology: This hospital-based cross-sectional study was conducted with a total of 420 study subjects. Examination of the eyes for REs was carried out using a refractometer. The REs were noted in myopia < -0.5 dioptres (D), hypermetropia > + 0.5 D, and astigmatism > 0.5 cylinder D. The data were statistically subjected to a statistics test. Categorical measurement was presented as frequency (percentage). The association between the parameters was done using the chi-square test. A p-value < 0.05 was considered statistically significant.

Results: Out of 420 examined, REs were present among 147 (35%) study subjects, and myopia 67 (16%) was the highest prevalent in comparison to hypermetropia 42 (10%) and astigmatism 38 (9%). The male subjects had 77 (34.4%) REs, and the female subjects had 70 (35.7%) REs. In both genders, myopia was the highest prevalent, followed by hypermetropia and astigmatism.

Conclusion: The prevalence of REs among children is alarming, and it should be corrected at an early age to prevent further complications in adulthood. Ophthalmologists should generate regional data about the prevalence of REs, create awareness about the prevention of REs among the risk population, and utilize government-sponsored blind eradication programs for comprehensive eye care in the larger interest of the affected population and risk population.

## Introduction

Refractive error (RE) is a condition in which the optical system of the non-accommodating eyes is not able to bring parallel rays of light to focus on the retina [1]. REs are the second major cause of blindness in India after cataracts. Over a quarter of outpatient attendance at eye clinics and hospitals is due to REs [2]. Population-based data on the prevalence of childhood blindness, which are needed to set priorities and plan strategies to reduce childhood blindness, are limited worldwide, including from India [3,4]. Nearly one in four blind persons suffers from REs, while three in four persons with low vision suffers from RE [5]. REs, if uncorrected, lead to impairment or decreased quality of life for millions of people worldwide, irrespective of their age, sex, and ethnicity [6]. The literature indicated that TV watching from a short distance, electronic device usage for a long time, use of dim light while reading, family history, gender (female), and near-work activity are major causes for REs [7-9]. In India, as of January 2017, there are 365 million children aged <15 years; therefore, providing vision screening for all children is cost-effective and daunting. Eye care facilities in the country vary between and within regions. School-based vision screening services are cost-effective in assessing correctable causes of decreased vision [10]. Region-specific prevalence assumes greater importance and is necessary for policy decisions. Hospital-based cross-sectional studies are more feasible. In previous studies, varied prevalences of different types of REs in children were investigated in different geographical areas. There is no such study undertaken on children between the ages of seven to 15 years in the Raichur district of Southern India. Hence, this cross-sectional study was conducted to evaluate the hospital-based prevalence of REs among the child population aged 7-15 years from the Raichur district of Karnataka state in Southern India.

## Materials and methods

Study settings, study population, and inclusion and exclusion criteria

This is a hospital-based cross-sectional study conducted among the children of age group seven to 15 years reported to the outpatient department of the hospital, which are the inclusion criteria of the study. The subjects with a past history of ocular surgery, trauma, spasm of accommodation, congenital anterior segment abnormalities, asthenopic complaints, and uncooperative children were excluded from the study.

Final sample size determination

In a pilot study, the prevalence of the condition of interest was found to be 28% out of 50 convenient pilot samples. For the cross-sectional study, the required sample size was calculated to ensure that the estimated prevalence in the population would be within 5% of the true prevalence with a 95% confidence interval. Using the standard formula for sample size calculation in prevalence studies, where the prevalence (P) is 0.28, the desired confidence level (Z) is 1.96, and the absolute precision (d) is 0.05, the sample size was determined as follows: n=Z2p (1-p)/d2; that is, n=(1.96)2 0.28(1-0.28)/(0.05)2=310 [11].

Sampling method and study period

The final sample required is 310 as per the sample size calculated. During the process of study undertaken between 15 September 2023 to 20 April 2024, approximately 1,130 children were reported between the ages of zero to 15 years, and we have done stratified random sampling. The children were divided into two groups: Group A with ages of 0-6 years and Group B with the ages of seven to 15 years. Only Group B was initially selected as age group strata, and from this group, the random final selection of study participants was made for the study based on the inclusion and exclusion criteria. Our set final sample was 310, but during the set study period, we got a total of 420 study subjects who met our criteria. Hence, they have all been included in our study as the final study sample.

Ethical consideration

The study had been approved by the Institutional Review Board (IRB) of Raichur Institute of Medical Sciences, Raichur, with no: RIMS/IEC/2023-24/18 (dated 31.07.2023). The children between the ages of seven and 12 have provided oral consent, followed by the signature of their parents on the consent form. We obtained written consent from the children between 13 and 15 years old with the signature of their parents on the form as per the Indian Council of Medical Research (ICMR) guidelines for children's consent in research protocol [12].

The selected study population and their parents were explained about the study objectives and protocol. The participants were allowed to withdraw from the research at any time without giving any explanations to the investigators. Investigators have maintained the confidentiality of the individual research data.

Operational definition and examination of eyes for REs

The definitions used for the analysis were the following: myopia was defined as spherical equivalent (SE) < -0.5 diopters (D), hypermetropia was defined as SE more than +0.5 D, and astigmatism > 0.5 cylinder D [13]. During our study time, the parents of the 7-15 years age group were offered to fill out the pretested questionnaire. The demographic data (age, gender, year of study) and the past ocular history (surgery, trauma, with spasm of accommodation, congenital anterior segment abnormalities, asthenopic complaints) were included in the questionnaire; after the assessment of the responses, 420 child participants without a history of ocular problems have been considered as study participants.

The examination of the eyes to detect REs was as follows: Streak retinoscopy was the instrument adopted to detect spherical refraction (hypermetropia and myopia) and cylindrical refraction (astigmatism). Study subjects' eyes were kept open for the relaxation of accommodation, and their eyes were dilated with 1% of one drop of cyclopentolate hydrochloride. A calm environment was maintained, and dim light was provided for better contrast of the papillary reflex. The study participant was seated upright, and the arm-length gap was maintained between the operator and the study participant. The direction of the streak retinoscope had been turned at the right eye of the study participant for proper visualization of the red reflex. The retinoscope had been swept horizontally across the study subject’s pupil. The movement of the reflex in the pupil was compared to moving the retinoscope. The retinoscope streak was rotated to the horizontal position and swept vertically across the pupil. With the rotation of the streak retinoscope, the sweeping was repeated for the oblique meridians at 45 and 135 degrees. For the detection of spherical errors, the observation of reflex had consistency in direction, brightness, speed, and width in all meridians. For the determination of astigmatic errors, the reflex in different meridians appeared differently [14,15]. Determination of the spherical component was done by achieving neutrality with the correcting lenses. Different lenses were held in front of the examined right eye. The reflex filled the entire pupil without perceived movement; after the operator swept across the pupil in this way, a neutral point had been achieved. Rechecking the neutral point was done by observing the reflex movement when it is moved forward and backward from the normal working distance [15,16].

The left eye of study participants was examined in a similar method; the investigator held the instrument in their left hand and using their left eye looked through the retinoscope.

Interpretation of REs: For the detection of final spherical errors, the working distance has been used by the operator to calculate the number of dioptres that needed to be offset (the inverse working distance in meters from the retinoscope had been subtracted). If the working distance between the lens and retinoscope was 200 cm, then 1/1 m (0.5 D) had been subtracted from the retinoscope to obtain the spherical error. The sphero-cylindrical corrections minus working distance corrections have been recorded as the results. The first number depicts spherical power (in dioptres); plus power is a representation of hypermetropia, and minus power is an indication of myopia. Astigmatism is represented by the second and third numbers; the second number is cylinder power, and the third number is the axis that the cylinder was neutralized [17].

A single calibrated operator examined all study subjects with the same instrument.

Data analysis

Statistical analysis was performed using IBM SPSS Statistics for Windows (version 25.0; IBM Corp., Armonk, NY). The categorical measurements were presented as frequency (percentage). The association between the parameters was done using the chi-square test. A p-value less than 0.05 was considered statistically significant.

## Results

Out of 420 subjects observed, 147 (35%) were found to have REs; among the REs, myopia was highest at 67 (16%), followed by hypermetropia at 42 (10%) and astigmatism at 38 (9%). In terms of gender, females have the highest REs in 70 (35.7%) subjects out of 224 compared to males in 77 (34.4%) participants out of 196, and myopia was the highest prevalent in both males and females compared to other REs. No significant association is observed between the REs and gender (p>0.05) (Table 1).

**Table 1:** Refractive errors and gender *Significant (no significant association was observed between the refractive errors and gender, i.e., p>0.05).

Refractive error	Total participants (N=420)	Total male participants (n=224)	Total female participants (n=196)	P value (<0.05 is significant)
Myopia, n (%)	67 (16)	36 (16.1)	31 (15.8)	0.943
Hypermetropia, n (%)	42 (10)	22 (9.8)	20 (10.2)	0.896
Astigmatism, n (%)	38 (9.0)	19 (8.5)	19 (9.7)	0.666
REs total, N (%)	147 (35)	77 (34.4)	70 (35.7)	

As per age-wise comparison in each group, myopia was the highest prevalent in all ages, except in age group 12; in the age group of 12 years, myopia and hypermetropia were equally distributed and higher compared to astigmatism, and there was no significant association. In gender-wise and age-wise comparison, in all-female population age groups, myopia was more prevalent, except in age group 7 and age group 12. In age group 7, myopia, hypermetropia, and astigmatism were equally distributed. In age group 12, myopia and hypermetropia were equally distributed and higher than astigmatism. In the male population in all age groups, myopia was more prevalent compared to hypermetropia and astigmatism, except for age group 12. In age group 12, myopia and hypermetropia are equally distributed and higher compared to astigmatism. There was no significance between age group and gender-wise comparisons (Table 2).

**Table 2:** Refractive errors with gender- and age-wise comparisons *Significant (no significant association was observed between the refractive errors with age and gender, i.e., p>0.05).

Refractive error	Gender	Age (Years)									Total (N=420) N (%)	P value (<0.05 is significant)
		7 (N=43) N (%)	8 (N=38) N (%)	9 (N=35) N (%)	10 (N=40)N (%)	11 (N=31) N (%)	12 (N=37) N (%)	13 (N=70) N (%)	14 (N=48) N (%)	15 (N=78) N (%)		
Myopia	Female	2 (4.7)	4 (10.5)	4 (11.4)	3 (7.5)	3 (9.7)	4 (10.8)	4 (5.7)	4 (8.3)	3 (3.8)	31 (7.4)	0.996
	Male	4 (9.3)	3 (7.9)	3 (8.6)	5 (12.5)	3 (9.7)	3 (8.1)	5 (7.1)	3 (6.3)	7 (9.0)	36 (8.6)	0.857
	Total	6 (14)	7 (18.4)	7 (20)	8 (20)	6 (19.4)	7 (18.9)	9 (12.9)	7 (14.6)	10 (12.8)	67 (16)	0.984
Hypermetropia	Female	2 (4.7)	2 (5.3)	2 (5.7)	2 (5)	2 (6.5)	4 (10.8)	2 (2.9)	2 (4.2)	2 (2.6)	20 (4.8)	0.991
	Male	2 (4.7)	2 (5.3)	2 (5.7)	3 (7.5)	2 (6.5)	3 (8.1)	3 (4.3)	2 (4.2)	3 (3.8)	22 (5.2)	0.999
	Total	4 (9.3)	4 (10.5)	4 (11.4)	5 (12.5)	4 (12.9)	7 (18.9)	5 (7.1)	4 (8.3)	5 (6.4)	42 (10.0)	0.999
Astigmatism	Female	2 (4.7)	3 (7.9)	3 (8.6)	1 (2.5)	2 (6.5)	2 (5.4)	2 (2.9)	2 (4.2)	2 (2.6)	19 (4.5)	0.995
	Male	2 (4.7)	2 (5.3)	2 (5.7)	2 (5.0)	1 (3.2)	2 (5.4)	2 (2.9)	2 (4.2)	4 (5.1)	19 (4.5)	0.970
	Total	4 (9.3)	5 (13.2)	5 (14.3)	3 (7.5)	3 (9.7)	4 (10.8)	4 (5.7)	4 (8.3)	6 (7.7)	38 (9.0)	0.987

## Discussion

In the present study, the prevalence of RE was 147 (35%) out of 420 observed participants. Pavithra et al. found REs in 97 (7.03%) school children out of 1,378 among 7-16 years old at Rajarajeshwari Medical College, Bangalore, in India. Among them, myopia was found in 61 (4.4%) subjects, followed by astigmatism and hypermetropia, which were detected in 14 (1.03%) and 22 (1.6%) participants, respectively [18]. Prema et al. observed REs in 192 (30.57%) school children aged 12 years out of 628 children at selected four schools in the Kancheepuram district of South India [19]. Bangal et al. found REs in 82 (16.4%) participants in the western part of Maharashtra state of South India among 500 children between six and 16 years old. The most common RE was myopia as observed in 52 (63.41%) subjects, followed by astigmatism in 18 (21.95%) and hypermetropia in 12 (14.63%) participants [20].

In our current study, myopia in 67 (16%) subjects together in both genders was the highest prevalent in comparison to hypermetropia in 42 (10%) and astigmatism in 38 (9%) subjects. Srivastava et al. conducted a study among 2024 school-going children aged 6-15 years at the Institute of Medical Sciences, Banaras Hindu University, Varanasi city, North India, and found the overall prevalence of REs, together in urban and rural children in 353 (17.43%) study subjects. Myopia was the highest prevalence in both urban and rural children, which was detected in 153 (42.17%) participants in urban children and 90 (24.22%) participants in rural children [21].

Bakare et al. examined 3,054 school-going children aged 11-15 years of Pimpri Chinchwad Municipal Corporation in India and found 368 (12.04%) children with uncorrected REs. Myopia was found in 204 (6.8%) participants, followed by astigmatism in 148 (4.8%) and hypermetropia in 16 (0.53%) subjects [22]. Basu et al. also reported 457 (15.22%) prevalences of RE out of 3,002 participants; myopia was found in 418 (91.47%), followed by hypermetropia in 21 (4.6%) and astigmatism in 18 (0.04%) [23]. Bhutia et al. investigated 15,954 children aged 14-17 years and found REs in 1,069 (6.7%) subjects; myopia was most commonly observed RE in 335 (31.1%) children, followed by astigmatism in 317 (29.4%) participants and hypermetropia in 29 (2.6%) subjects in East Sikkim of Northeast India [24].

In our study, out of 224 male participants, 77 (34.4%) have REs, and out of 196 female study subjects, 70 (35.7%) have REs. Singh et al. also found REs in 287 (34.91%) males out of 822 observed and in 257 (35.55%) females out of 723 examined [25]. This study is important because it throws light on the prevalence of REs among children between seven and 15 years of age. About 13% of the Indian population is in the age group of 7-15 years, and about 20% of children develop REs by the age of 16 years [26].

As per the National Program for Control of Blindness and Visual Impairment (NPCBVI), the first goal is to reduce the backlog of blindness through the identification and treatment of blinds at primary, secondary, and tertiary levels based on the assessment of the overall burden of visual impairment in the country. Under this program, the central government and state governments provide grants-in-aid for comprehensive eye healthcare for adults and children, and the program also includes the promotion of research and surveys [27].

In 1983, the national health policy of India reiterated that blindness was an important public health problem and set a target to reduce the blindness prevalence from 1.4% to 0.3% [28].

Till a few years ago, the national program for the control of blindness was a cataract-centred program. However, currently, there is funding for the management of diabetic retinopathy, glaucoma, ocular trauma, childhood blindness, keratoplasty, squint, low vision, and retinopathy of prematurity [29].

This is a hospital-based study, so it is economical and feasible to conduct a study, unlike the field study of enrolling school children in schools and community camps.

Limitations

The study, as a hospital-based cross-sectional study, might have not covered wider populations, and the study has been done on a particular period and on outpatients of the hospital. Hence, it may not reflect the entire population, and sometimes if the streak retinoscope was kept off the axis, it might have produced spherical errors.

The following are the recommendations:

(1) The burden of vision impairment should be reduced due to REs, and it is necessary for early diagnosis of the children and to take remedial measures to correct REs.

(2) The risk factors and risk population should be identified, and the risk population should be educated on the protection of their eyes using protective eyewear, sunglasses, and regular checkups.

(3) Ophthalmologists should create awareness among other health workers and the public about the availability of various preventive measures, cost-effective interventions, and government health policies for blindness control at the district level.

(4) Ophthalmologists should update their current knowledge, enhance their skills, and promote research for the optimal care of the eyes.

## Conclusions

Our findings pointed to the high prevalence of REs among children of seven to 15 years and estimated that myopia was highly prevalent. Our study indicated the high impact of particular types of REs on all age groups and genders. The key point is that our study was conducted at a government teaching hospital using a streak retinoscope, which is feasible, economical, and less manpower required. REs among children should be detected and corrected at an early age in order to prevent visual impairment and blindness. Our findings highlight the importance of regional research data, which will aid in national statistics to launch new government healthcare programs. This research data can also be utilized by government blindness eradication programs operating at a district level to specifically focus on the affected and risk population.
